# Immunodeficiencies in Adults: Key Considerations for Diagnosis and Management

**DOI:** 10.1007/s12016-025-09103-9

**Published:** 2025-10-17

**Authors:** Jean Regina, Jacqueline Doms, Eleftheria Kampouri, Christel Gerber, Oriol Manuel, Pierre-Alexandre Bart, Fabio Candotti, Denis Comte

**Affiliations:** 1 Service of Internal Medicine, Department of Medicine, Lausanne University Hospital (CHUV) and University of Lausanne, Lausanne, Switzerland; 2 Service of Immunology and Allergy, Department of Medicine, Lausanne University Hospital (CHUV) and University of Lausanne, Lausanne, Switzerland; 3 Service of Infectious Diseases, Department of Medicine, Lausanne University Hospital (CHUV) and University of Lausanne, Lausanne, Switzerland; 4 Transplantation Center, Lausanne University Hospital (CHUV) and University of Lausanne, Lausanne, Switzerland

**Keywords:** Primary immunodeficiencies, Secondary immunodeficiencies, Inborn errors of immunity, Common variable immunodeficiency

## Abstract

Immunodeficiencies in adults are increasingly recognized yet often remain underdiagnosed, leading to significant morbidity from recurrent infections, autoimmunity, and malignancy. Both primary immunodeficiencies (PIDs), now known as inborn errors of immunity (IEI), and secondary immunodeficiencies (SIDs) contribute to immune dysfunction in adults. Although SIDs are more common in adults due to factors like medications, malignancies, metabolic disorders, chronic conditions, and protein-losing conditions, IEI—particularly common variable immunodeficiency (CVID)—can also manifest in adulthood with diverse clinical features. Early recognition is crucial, with key warning signs including recurrent sinopulmonary infections, unexplained autoimmunity, poor vaccine responses, chronic diarrhea, bronchiectasis, and persistent lymphadenopathy. The diagnostic approach should be systematic. It begins with a detailed patient history and status followed by the evaluation of immunoglobulin levels, lymphocyte subsets, vaccine-specific antibody responses, and exclusion of secondary causes. Genetic testing, increasingly accessible, plays an important role in confirming the diagnosis of IEI and guiding prognosis and treatment. Management strategies focus on treating the underlying condition in SIDs. Preventive measures, including antimicrobial prophylaxis, vaccination, and immunoglobulin replacement therapy (IGRT) in patients with significant antibody deficiencies, are essential for reducing infections and complications in high-risk patients. Given the growing recognition of adult-onset immunodeficiency, clinicians should maintain a high index of suspicion and adopt a structured diagnostic and management approach to improve patient outcomes and quality of life.

## Introduction

Immunodeficiency, defined as a weakened or dysfunctional immune system that increases susceptibility to infections and cancer, is common in adults. Recent estimates suggest that more than 6% of US adults may be affected [1], with most cases resulting from secondary immunodeficiencies (SID). These deficiencies arise due to external factors such as human immunodeficiency virus (HIV) infection, medications, intrinsic factors including systemic diseases, malignancies, and metabolic disorders, or a combination of both. Primary immunodeficiencies, traditionally referred to as “primary immune deficiencies (PID),” are now classified as inborn errors of immunity (IEI). These conditions can also be diagnosed in adult patients, often with delayed diagnoses due to their heterogeneous manifestations. Importantly, this terminology can be misleading, as not all IEI initially present with immunodeficiency, and some may manifest only in adulthood [2–5]. Moreover, IEI encompasses not only classical primary immunodeficiencies with recurrent or severe infections, but also conditions of immune dysregulation, including autoimmunity, autoinflammation, allergy, and malignancy.

This review aims to provide clinicians with a practical framework for recognizing immunodeficiency in adults, with a focus on SID, which accounts for most cases. We will outline key warning signs, initial diagnostic steps, and indications for specialist referral and management. Additionally, we will discuss common variable immunodeficiency (CVID), the most frequently diagnosed symptomatic IEI in adults. These considerations are based on a narrative review of literature, expert consensus, and the authors’ clinical experience to guide decision making in practice.

## Methods

This narrative review searched PubMed and Scopus from database inception to May 1, 2025, without language or date restrictions. The search terms included *immunodeficiency*,* common variable immunodeficiency*,* primary immunodeficiency*,* inborn errors of immunity*,* recurrent infections*,* opportunistic infections*,* and antibiotic prophylaxis*. We also reviewed key American and European guidelines on immunodeficiency, infectious risk stratification, treatment, and vaccination.

## Clinical Presentation and Warning Signs

The clinical presentation of immunodeficiencies is highly variable, but most patients present with recurrent and/or severe infections. While no universally accepted definition exists for recurrent or severe infections in the context of immunodeficiency, certain patterns of infections should raise suspicion of an underlying immune defect. These include opportunistic infections (i.e., infections due to pathogens that would not cause disease in individuals with healthy immune systems), recurrent sinopulmonary infections, and severe infections requiring intravenous antibiotics, hospitalization, invasive interventions or that are life-threatening [6]. In the context of IEI, the European Society for Immunodeficiency (ESID) and the Jeffrey Modell Foundation (JMF) have proposed warning signs [7, 8] which should raise the suspicion of an underlying IEI in adults and children (Table 1). The JMF criteria have been clinically validated to evaluate IEI and SID patients [9], and may serve as a useful tool for the early detection of SID. Other warning signs for immunodeficiency in adults that should not be overlooked include chronic diarrhea, bronchiectasis, poor vaccine responses, autoimmune disorders, persistent lymphadenopathy or splenomegaly, and granulomatous lesions [10, 11]. Additional scoring systems for early identification of patients affected by IEI based on expert opinions are in development, aiming to improve diagnostic accuracy in primary care settings [12]. Data-driven model systems using machine learning can also help screen individuals at high risk of IEI [13].

**Table 1:** European Society for Immunodeficiency (ESID) and the Jeffrey Modell Foundation (JMF) warning signs for primary immunodeficiency in adults

The 6 ESID warning signs (≥ 1 criterion)	The Jeffrey Model Foundation warning signs (≥ 2 criteria)
1. Four or more infections requiring antibiotics within one year (otitis, bronchitis, sinusitis, pneumonia) 2. Recurring infections or infections requiring prolonged antibiotic therapy 3. Two or more severe bacterial infections (osteomyelitis, meningitis, septicemia, cellulitis) 4. Two or more radiologically proven pneumonia within 3 years 5. Infection with unusual localization or unusual pathogen 6. A family history of primary immunodeficiency	1. Two or more new ear infections within one year 2. Two or more new sinus infections within one year, in the absence of allergy 3. One pneumonia per year for > 1 year 4. Chronic diarrhea with weight loss 5. Recurrent viral infections (colds, herpes, warts, condyloma) 6. Recurrent need for intravenous antibiotics to clear infections 7. Recurrent, deep abscesses of the skin or internal organs 8. Persistent thrush or fungal infection on skin or elsewhere 9. Infection with normally harmless tuberculosis-like bacteria 10. A family history of primary immunodeficiency

Adapted from references (2,3)

## Types of Immunodeficiency

### Primary Immunodeficiency

Distinguishing between IEI and SID in adult patients can be challenging, as autoimmune and hematologic or oncologic conditions can be both the causes and the manifestations of immune dysfunction. IEI is usually diagnosed during childhood, but some entities, particularly CVID, may manifest in adulthood. In addition, hypomorphic variants of classically severe IEI (e.g., mutations in RAG1/2, ADA2, or NF-κB1 genes) can present with attenuated phenotypes and be diagnosed later in life [14]. CVID is defined by reduced levels of immunoglobulins G (IgG) along with low levels of IgA and/or IgM, defective responses to immunizations, and abnormal B cell immunophenotype, often with significant reduction of isotype-switched memory B cells [15]. CVID represents a heterogenous group of disorders with variable clinical phenotypes, contributing to delayed diagnosis. Most patients are diagnosed between 20 and 45 years of age after multiple consultations and hospitalizations [16]. While genetic variants are identified in 25–30% of cases, novel disease-causing mutations continue to be discovered [17]. The clinical presentation and potential complications can vary according to the underlying genetic defect.

The main clinical feature of CVID is recurrent respiratory tract infections, such as pneumonia, sinusitis, and otitis media, mainly due to *Streptococcus pneumoniae* and *Haemophilus influenzae*, which have been reported in over 90% of patients in large studies [16]. Viral (e.g., herpes zoster), fungal (e.g., *Pneumocystis jirovecii*), and parasitic (e.g., *Giardia*) infections are less frequent [16]. In adults with recurrent respiratory infections, the presence of allergy, autoimmunity, and/or granulomatous disease should raise the suspicion of underlying CVID [18]. Patients with predominant non-infectious complications (autoimmunity, lymphoproliferation, enteropathy) are increasingly recognized as a distinct subgroup of CVID with different pathophysiology and outcomes compared to those with mainly infectious manifestations [19]. Additional complications affecting CVID patients include atopy, immune thrombocytopenic purpura, hemolytic anemia, lymphoproliferation, splenomegaly, protein-losing enteropathy, and granulomatous lymphocytic interstitial lung disease (GLILD) [20]. Bronchiectasis and interstitial lung disease are major complications of pulmonary infections and represent key signs for CVID diagnosis.

### Secondary Immunodeficiency

SID conditions are acquired, tend to appear later in life, and can be transient or permanent. They result from external factors that impair immune function. Identifying the underlying cause is crucial, as targeted interventions can often alleviate or reverse immune dysfunction.

#### SID Inducing Drugs

Medications represent one of the most frequent causes of SID in adults. Various drug classes can impair the immune system, including cytotoxic agents, glucocorticoids, traditional immunosuppressants, biological and targeted therapies, and cellular therapies, as well as certain antiepileptic and antipsychotic drugs (Table 2). A comprehensive review of current and past medication history is crucial for the recognition of drug-induced immunosuppression.

**Table 2:** Most common medications causing secondary immune deficiency

Medication family	Examples
B cell targeting monoclonal antibodies [21]	Rituximab, daratumumab, belimumab
Immunosuppressants and cytotoxic agents [22–24]	Prednisone and derivates, methotrexate, cyclophosphamide, mycophenolate mofetil, cyclosporin, calcineurin inhibitors, purine analogs
Tumor necrosis factor alpha inhibitors [25]	Infliximab, etanercept
Tyrosine kinase inhibitors [26]	Ruxolitinib, tofacitinib, baricitinib
Others [27–30]	Anticonvulsivants: carbamazepine, phenytoin, lamotrigine Antipsychotics: clozapine Antimalarials: chloroquine and hydroxychloroquine

Glucocorticoids exert anti-inflammatory and immunosuppressive effects in a dose and duration-dependent manner [31], and may lead to hypogammaglobulinemia and CD4 lymphopenia [22, 32]. Similarly, biological agents used to treat autoimmune disorders and malignancies increase the risk of infection through mechanisms specific to their targets and off-target enzyme inhibitions. For example, anti-CD20 monoclonal antibodies, such as rituximab, cause prolonged B-cell depletion, while hypogammaglobulinemia can also occur, especially with prolonged courses [33]. Some patients also develop late-onset neutropenia, which is believed to result from immune dysregulation, including cytokine imbalance that impairs neutrophil production [34]. Impaired vaccine responses, severe respiratory infections, reactivation of hepatitis B virus, and even progressive multifocal leukoencephalopathy (PML) in rare cases are reported [33]. Tumor necrosis factor alpha (TNF-α) inhibitors are consistently associated with an increased risk of tuberculosis and potentially a higher risk of serious infections, particularly early in treatment, as well as with reactivation of hepatitis B infection [25].

Of note, immunosuppressive drugs are often used in combination, leading to an increased immunosuppressive state. For example, combined drug regimens used after solid organ transplantation (SOT) to avoid transplant rejection often include glucocorticoids, calcineurin inhibitors, lymphocyte proliferation inhibitors and/or mechanistic target of rapamycin (mTOR) inhibitors, while anti-thymocyte globulins (ATG) and anti-IL-2 receptor antibodies are also frequently used at induction. These agents are known to cause profound immunosuppression, especially of the cell-mediated immunity and lead to a high infection risk after SOT [35].

The list of biological and targeted molecules with immunosuppressive potential is rapidly expanding, reflecting the continuous development of novel agents in oncology, autoimmunity, and transplantation. It is increasingly challenging for non-specialists to remain familiar with their diverse mechanisms of action and infectious risks. For this reason, updated consensus documents, such as those from the ESCMID Study Group for Infections in Compromised Hosts (ESGICH), are valuable resources that provide comprehensive, regularly revised guidance on infection risk stratification and management [36].

#### Metabolic Causes

Diabetes mellitus is the most common metabolic disorder associated with immune dysfunction, leading to an increased susceptibility to infections [37]. Chronic hyperglycemia impairs both innate and adaptive immunity, affecting neutrophil chemotaxis, phagocytosis, and cytokine responses. Obesity without diabetes is also associated with autoimmunity and immune dysfunction, suggesting a causal relationship [38]. Patients with Cushing’s disease are at a higher risk of infection due to increased endogenous glucocorticoid production, which suppresses inflammatory and immune responses [39]. To a lesser extent, thyroid dysfunction can contribute to weakening immune function due to its effect on T-cell function. Other hormonal dysfunctions that can impact the immune system include hypopituitarism, adrenal insufficiency, growth hormone deficiency, and imbalances in sex hormones.

#### Infectious Causes

Chronic infections can contribute to SID by directly targeting immune cells or inducing prolonged immune activation and exhaustion. HIV is the most well-known infectious cause. In the absence of antiretroviral therapy, HIV infection progressively leads to CD4 + T-cell depletion and impaired cell-mediated immunity, increasing vulnerability to encapsulated bacteria (*S. pneumoniae*) and severe opportunistic infections including viral (reactivation of herpesviruses, JC virus), fungal (including *Pneumocystis jirovecii*, *Cryptococcus *spp, *Candida *spp), parasitic (toxoplasmosis), and mycobacterial pathogens. Infection risk is strongly correlated with CD4 T-cells counts. HIV also disrupts innate immunity, impairing alveolar macrophage function, reducing neutrophil and NK cell activity, and altering B-cell responses, leading to defective antibody production and poor vaccine-induced immunity [40, 41]. Additionally, chronic immune activation and gut barrier disruption contribute to systemic immune dysfunction.

#### Protein Loss

Protein-losing conditions can lead to hypogammaglobulinemia due to excessive immunoglobulin loss that is not adequately compensated by synthesis. In nephrotic syndromes, proteins including immunoglobulins are lost in the urine, with hypogammaglobulinemia further exacerbated by impaired IgG synthesis and by the use of B-cell-targeting medications [42, 43]. Protein-losing enteropathy (PLE) results from excessive protein loss through the gastrointestinal tract and can complicate various conditions, including inflammatory bowel disease, systemic lupus erythematosus, coeliac disease, graft versus host disease, bacterial overgrowth, and intestinal infections such as viral or parasitic enteritis and Whipple disease. PLE may also be associated with cardiovascular disorders and neoplasms [44]. Additionally, PLE can occur as a complication of IEI, such as CVID, further exacerbating hypogammaglobulinemia.

#### Lymphatic Malformations and Primary Intestinal Lymphangiectasia

Lymphatic malformations and primary intestinal lymphangiectasia (PIL) are rare but significant causes of SID. These conditions are characterized by abnormal development or dysfunction of lymphatic vessels, leading to excessive loss of lymphatic fluid rich in proteins, lymphocytes, and immunoglobulins [45, 46]. As a result, affected individuals often develop hypogammaglobulinemia, lymphopenia, and impaired humoral and cellular immune responses, increasing their susceptibility to infections. Clinically, it manifests with peripheral edema, chylous ascites, chronic diarrhea, and recurrent infections, particularly with encapsulated bacteria [47]. Patients with PIL may also present with profound and selective lymphopenia, further compromising immune defenses [48]. Generalized lymphatic malformations, as seen in certain congenital syndromes (e.g. Hennekam syndrome), can also lead to SID due to systemic lymphatic leakage [49, 50].

#### Hematological Malignancies

Patients with hematological malignancies have a high infectious risk due to the underlying malignancies and treatments. Infectious risk is highest in those with acute leukemia receiving induction and consolidation chemotherapies resulting in prolonged neutropenia (> 10 days), and in recipients of allogeneic hematopoietic cell transplantation (HCT), especially in the context of additional immunosuppression for prevention or management of graft versus host disease (GVHD) [51, 52]. Novel cellular therapies such as chimeric-antigen-receptor (CAR) T-cell therapies are increasingly used in B-cell and plasma cell malignancies and lead to profound and persistent immune deficits in cellular and humoral immunity [53]. Importantly, emerging treatments—including bispecific T-cell engagers and CAR T-cell therapies—further underscore the need for heightened vigilance in assessing infection risk. The duration of neutropenia is a key determinant of infection risk in these “high risk” patients with hematological malignancies, increasing risk for bacterial and fungal infection and dictating preventive strategies. Cellular immunodeficiency after allogeneic HCT increases the risk of herpesvirus infection, especially cytomegalovirus (CMV), that requires special prophylactic strategies in this setting. Finally, humoral immune deficits are also frequent, both after HCT and CAR T-cell therapies (“on-target off-tumor effects”), and lead to frequent sinopulmonary infections.

Other hematological malignancies, such as myeloma and lymphoma, can lead to immunodeficiencies through multiple mechanisms [54]. Multiple myeloma disrupts normal antibody production through the expansion of clonal plasma cells that produce large quantities of dysfunctional monoclonal immunoglobulins, while simultaneously suppressing normal immunoglobulin synthesis. In lymphoma, immune dysfunction arises due to bone marrow involvement, chemotherapy-induced myelosuppression, and direct impairment of B- and T-cell function. Patients with these malignancies are at increased risk for recurrent bacterial infections, particularly with encapsulated organisms, due to impaired humoral immunity.

Hypogammaglobulinemia is frequent in patients with chronic lymphocytic leukemia (CLL) due to the progressive loss of normal B-cell function, leading to impaired immunoglobulin production and defective humoral immunity [55]. The severity of B-cell dysfunction correlates with disease progression and treatment exposure, particularly with anti-CD20 monoclonal antibodies and Bruton tyrosine kinase (BTK) inhibitors, which further deplete B-cell populations.

Thymoma can also be associated with B-cell lymphopenia and hypogammaglobulinemia, leading to a rare form of secondary immunodeficiency known as Good’s syndrome [56]. The immune defect in Good’s syndrome extends beyond B-cell deficiency and may include T-cell abnormalities, resulting in susceptibility to both bacterial and opportunistic infections.

#### Malnutrition and Aging

Malnutrition is a leading cause of SID worldwide and affects both children and adults, particularly in low-income countries. Chronic caloric and protein deficiencies impair immune cell development, proliferation, and function, increasing susceptibility to infections. Micronutrient deficiencies, including zinc, vitamin A, vitamin D, iron, and selenium, weaken mucosal barriers, and impair innate and adaptive immune responses [57]. Hypoproteinemia results in decreased T cell generation and function, with immunosuppression correlating with the severity of protein depletion [58].

Immunosenescence, the gradual decline of immune function with age, results in reduced naïve T-cell production, impaired B-cell responses, and diminished innate immune activity. Elderly individuals experience weakened vaccine responses, increased susceptibility to infections, and a higher prevalence of chronic inflammation (“inflammaging”), which contributes to immune dysregulation [57]. These age-related changes along with a higher burden of comorbidities make older individuals particularly vulnerable to pneumonia, influenza, and other infectious diseases, often with more severe outcomes.

#### Other Causes

Chronic kidney disease (CKD) and liver cirrhosis can also lead to SID by multiple mechanisms. In CKD, uremic toxins impair neutrophil chemotaxis, phagocytosis and oxidative burst, reducing innate immune defenses and increasing susceptibility to bacterial infections [59]. Furthermore, CKD leads to T-cell dysfunction, with decreased activation, proliferation, and cytokine production, further compromising adaptive immunity [60]. Liver cirrhosis results in the dysfunction of Kupffer cells, the resident macrophages of the liver, resulting in impaired pathogen clearance and endotoxin tolerance. Additionally, it is associated with decreased acute-phase reactant production, impaired neutrophil function, and complement deficiency, all of which contribute to the increased risk of spontaneous bacterial peritonitis and systemic infections [61]. 

Other causes of SID include splenectomy, functional hyposplenia or asplenia, thymectomy, radiation therapy, and plasmapheresis. Splenectomy results in impaired clearance of encapsulated bacteria, predisposing individuals to overwhelming post-splenectomy infections syndrome (OPSI) [62]. Functional hyposplenia, as found in sickle cell disease patients, alters immunophenotype and also predisposes to bacterial infections [63]. Thymectomy, particularly when performed early in life, can significantly reduce T-cell output, leading to a weakened adaptive immune response [64]. Radiation therapy, especially when targeting the bone marrow or lymphoid tissues, leads to lymphopenia and neutropenia, increasing the risk of infections [65]. Plasmapheresis, used to remove pathogenic antibodies, can also deplete protective immunoglobulins [66].

## Workup for Suspected Immunodeficiency

When immunodeficiency is clinically suspected, investigations should begin with a detailed patient history and thorough physical examination. The history should assess recurrent, severe, or unusual infections, the age of onset, response to antibiotics or prophylactic treatments, and any history of chronic inflammatory, autoimmune, or allergic manifestations. A family history suggestive of IEI, the presence of comorbidities that could lead to SID, past and current medications, and prior immune evaluations must be reviewed. Finally, vaccination records and symptoms suggestive of hematologic malignancies or other occult neoplasia should be assessed. Physical examination should look for signs of chronic infections, autoimmune disorders, or systemic immune dysfunction, including splenomegaly, lymphadenopathy, malnutrition, signs of chronic lung or gastrointestinal diseases and skin abnormalities.

Investigations should be completed with a laboratory workup [6]. A reasonable, two-step approach is described in Table 3**.**

**Table 3:** Laboratory workup for clinically suspected immunodeficiency

Step 1	Step 2
• Full blood count^a^ • Serum level of immunoglobulin G, A and M and IgG subclasses^a^ • Renal and liver function tests • Albumin • CRP, ESR • Exclusion of chronic infections: HIV, HBV, HCV, tuberculosis • TSH and HbA1c • Serum protein electrophoresis and immunofixation • Nutritional assessment^CC^	• Lymphocyte subset analysis (at minimum T CD3 +, T CD4 +, T CD8 +, B and NK cells) • Specific antibody response to both protein/conjugated antigens (e.g. tetanus, diphtheria and *Haemophilus influenzae serotype b*) and polysaccharide antigens (e.g. *Streptococcus pneumoniae*) • CH50 (screening for classical complement pathway; AP50 if alternative pathway defect suspected)

Additional autoimmunity testing (e.g. Antinuclear Antibody (ANA), Extractable Nuclear Antigen (ENA)) can be considered when clinical suspicion of immune dysregulation exists but is not part of the systematic first-line evaluation

IgG subclasses interpretation requires correlation with clinical findings and vaccine-specific antibody responses

*CRP* C-reactive protein, *ESR* erythrocyte sedimentation rate, *TSH* Thyroid Stimulating Hormone, *HbA1c* glycated hemoglobin, *CD* cluster of differentiation, *NK* natural killer

^a^Repeat testing 4–6 weeks after acute infection

^CC^On a case-by-case basis

The first step, which can be conducted by a primary care physician, includes the evaluation of secondary causes, as well as of a full blood count and total serum immunoglobulin levels. Step two includes a more in-depth evaluation of cellular and humoral immunity, as well as the complement activity. This step should ideally be interpreted by an immunologist or immunodeficiency specialist. Investigations are tailored to each case and may include IgG subclass analysis, lymphocyte proliferation assay, neutrophil function test, and genetic screening. In specific situations, baseline lung functions and chest CT imaging should be considered to evaluate for interstitial lung disease, bronchiectasis, or thymoma.

Abnormal quantitative results for serum immunoglobulins or blood cell count may transiently occur during acute infection. Therefore, it is generally recommended to wait 4 to 6 weeks after the resolution of last acute infection before performing an immunodeficiency assessment. An unremarkable baseline biological workup does not exclude an underlying immunodeficiency, particularly in patients suffering from recurrent, severe, or unusual infections. If clinical suspicion remains high despite normal initial results, referral to an immunology specialist for further evaluation and long-term follow-up is warranted [6].

## Management of Immune Deficiency in Adults

The management of SID relies on two pillars: identifying and treating the underlying cause and preventing infections. The treatment of the etiology focuses on addressing the underlying disease process to reverse or slow down the progression of the immune deficiency whenever possible.

**Fig. 1:**
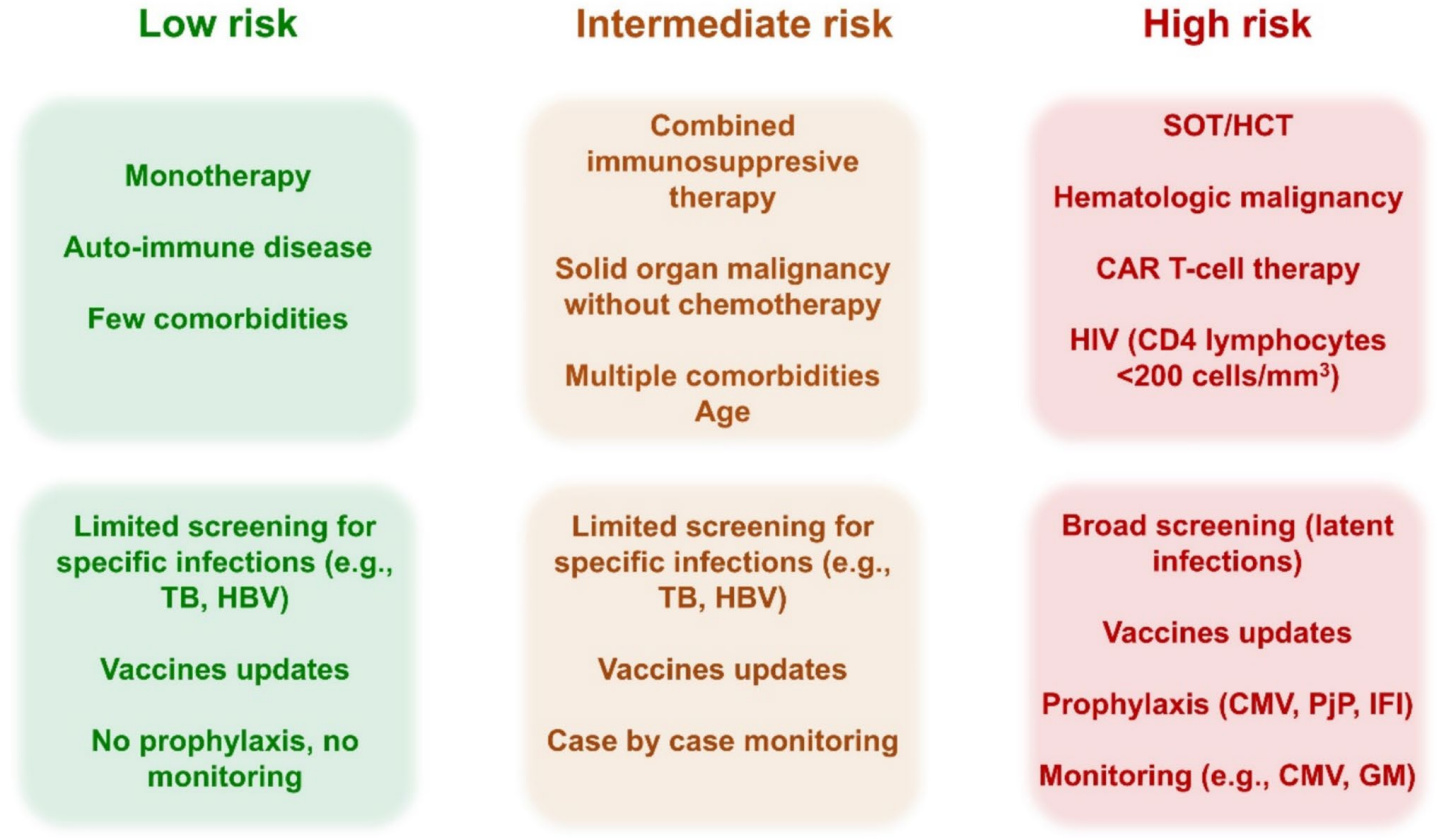
Conceptual framework for infectious risk stratification and preventive measures. Abbreviations: TB, tuberculosis; HBV, hepatitis B virus, SOT, solid organ transplant; HCT, hematopoietic cell transplantation; CAR T-cell, chimeric antigen receptor T-cells; HIV, human immunodeficiency virus; CMV, cytomegalovirus; PjP, *Pneumocystis jirovecii* pneumonia; IFI, invasive fungal infection; GM, galactomannan

The risk of infection varies significantly depending on the underlying condition, immunosuppressive treatment, and patient characteristics. Generally, infection risk is low in young patients without significant comorbidities receiving a single immunomodulatory agent for inflammatory diseases, while SOT and HCT recipients, patients with hematologic malignancies undergoing intensive chemotherapy, and patients with HIV infection and CD4 counts below 200 cells/mm^3^ are at the highest risk. Older patients with cancer and other comorbidities on combined immunosuppressive regimens are at intermediate risk. We propose a framework for infection risk stratification based on the patient, disease, and treatment characteristics (Fig. 1). Low-risk patients may benefit from targeted screening (e.g., latent TB in anti-TNF) and vaccination, while high-risk patients additionally require more extensive screening, antiviral and antifungal prophylaxis, and monitoring (e.g., CMV). In the following paragraphs, we outline general preventive measures applicable to all SID patients, as well as specific strategies tailored to different levels of infectious risk.

### General Preventive Measures

Prevention of infection primarily includes general hygiene measures, such as hand disinfection, wearing protective face masks during winter months in case of contact with crowds in closed spaces, and avoiding close contact with sick individuals [67].

Patients with SID require regular follow-ups with their primary care physician. Depending on their estimated infectious risk, they might also require follow-up with specialists to monitor immune function, infection risk, and treatment response. Follow-up visits should include clinical assessments, laboratory monitoring, and individualized adjustments to prophylactic or therapeutic strategies based on disease progression and emerging complications.

Early infection detection and intervention are crucial, as infections may present atypically, progress rapidly, and lead to severe complications. Patients should be educated on recognizing early signs of infection, including fever, persistent cough, shortness of breath, unexplained weight loss, recurrent diarrhea, or localized pain and swelling. Patients should be instructed to seek prompt medical attention if they develop any of these symptoms to facilitate early diagnosis and timely treatment.

### Antimicrobial Prophylaxis Based on Infectious Risk

Patients at high risk for infection (e.g., transplant recipients, cellular therapies recipients, HIV with CD4 counts below 200 cells/mm^3^) (Fig. 1) may require specific antimicrobial prophylaxis. *Pneumocystis jirovecii* prophylaxis is recommended in patients with cellular immunodeficiencies [68, 69]. First-line prophylaxis is trimethoprim–sulfamethoxazole (TMP-SMX), which also provides protection against *Toxoplasma gondii*. If TMP-SMX cannot be used, alternatives for *Pneumocystis jirovecii* include atovaquone or aerosolized pentamidine. Toxoplasmosis prophylaxis is also recommended in patients at high risk, including toxoplasma-seropositive HIV-infected persons with CD4 counts below 200 cell/mm^3^ and toxoplasma-seronegative heart transplant recipients receiving an allograft from a seropositive donor [69]. When TMP-SMX cannot be used, patients at risk for toxoplasmosis may require dapsone combined with pyrimethamine and folinic acid. Given their complexity and potential toxicities, they should preferably be prescribed with input from an infectious diseases or immunology specialist.

Other bacterial prophylaxis may also be required in specific setting. Long-term penicillin prophylaxis is recommended in patients with active graft versus host disease (GVHD) after allogeneic HCT due to increased susceptibility to recurrent bacterial infections with encapsulated bacteria [70]. Penicillin prophylaxis is recommended for patients who have not been vaccinated against *Neisseria meningitidis* and are treated with the anti-C5 antibody eculizumab. The optimal duration of prophylaxis, and its necessity in vaccinated individuals, remains a matter of debate [71, 72].

Antifungal prophylaxis with fluconazole or mold-active azoles (posaconazole) is warranted during periods of prolonged severe neutropenia in patients with hematologic malignancies receiving intensive chemotherapies and/or allogenic HCT [73]. A preemptive approach based on monitoring of galactomannan and early work-up with imaging and initiation of antifungal therapy in patients with persistent neutropenic fever can also be implemented [74]. Antiviral prophylaxis against HSV and VZV is also generally recommended in high-risk patients, particularly those with cellular immunodeficiencies [75]. CMV prophylaxis (letermovir, (val)ganciclovir) or or CMV monitoring with blood PCR and preemptive therapy at detection above certain viral-load thresholds is generally reserved for transplant recipients at risk [76, 77]. Prophylaxis for hepatitis B virus (HBV) may be needed in certain high-risk patients (e.g., patients with HBsAg + chronic infection and treated with rituximab, prolonged corticosteroid therapy, or chemotherapy) [78, 79]. Treatment of latent tuberculosis infection (LTBI), should generally be considered, especially for patients receiving TNF-α inhibitors, prolonged corticosteroid therapy, or chemotherapy [80].

### Vaccines

Vaccination is crucial for reducing infection risk, morbidity, and mortality in patients with SID. Due to the heterogeneous nature of SID and limited clinical data, vaccination recommendations are mainly based on expert opinion [81].

To maximize efficacy, vaccines must be administered as soon as immune deficiency is diagnosed or before initiating immunosuppressive therapy. Live attenuated vaccines, such as measles/mumps/rubella (MMR), live-attenuated influenza, varicella, and yellow fever vaccines should generally be avoided in immunocompromised individuals and, in some cases, by their household members, due to the risk of uncontrolled replication of the weakened pathogen in immunocompromised individuals. The immunogenicity of non-live vaccine is considered to be suboptimal in immunocompromised patients. Revaccination after receipt of HCT, SOT and cellular therapies is recommended. The quality of vaccine response in SID patients is often suboptimal, depending on the underlying disease. For this reason, household members, close contacts, and caregivers should also be vaccinated to reduce exposure risk.

Annual seasonal influenza and Covid-19 vaccinations are recommended in the fall/winter. For pneumococcal infections, the conjugate pneumococcal vaccine (preferably 20-valent) is recommended. Recombinant adjuvanted zoster vaccine is recommended in all VZV-seropositive individuals. Additional vaccinations may be recommended depending on immune deficit (e.g., *Neisseria meningitidis* in asplenic patients or patients treated with eculizumab) and exposures. Assessing the immune response by measuring vaccine-specific anti-pneumococcal antibodies can help to estimate protection level and monitor changes over time. Other vaccination strategies depend on the specific underlying disease or treatment and are outlined in Table 4.

**Table 4:** Vaccination suggestions according to the secondary immunodeficiency etiology

Condition	Recommended vaccinations
Liver cirrhosis [82]	- Annual Influenza and Covid-19 - Pneumococcal PCV20 - Hepatitis A upon diagnosis - Hepatitis B upon diagnosis - Tetanus, Diphtheria, Pertussis (Tdap) if not up to date
Anatomical or functional asplenia (including sickle cell disease) [83]	- Annual Influenza and Covid-19 - Pneumococcal PCV20 *- Haemophilus influenzae* b: one dose - Meningococcal (serogroups A, C, W, Y + serogroup B) - Tetanus, Diphtheria, Pertussis (Tdap) if not up to date
Chronic kidney disease [84]	- Annual Influenza and Covid-19 - Pneumococcal PCV20 if clearance < 30 ml/min or worsening (stages 4–5, National Kidney Foundation) - Recombinant zoster vaccine from age 50 and in end-stage disease (stages 4–5, National Kidney Foundation) or dialysis - Hepatitis B before hemodialysis initiation - Tetanus, Diphtheria, Pertussis (Tdap) if not up to date
Nephrotic syndrome [85]	- Annual Influenza and Covid-19 - Pneumococcal PCV20 - Tetanus, Diphtheria, Pertussis (Tdap) if not up to date
Diabetes with impact on renal function or cardiovascular disease [86]	- Annual Influenza and Covid-19 - Pneumococcal PCV20 if clearance < 30 ml/min or worsening (stages 4–5, National Kidney Foundation) - Recombinant zoster vaccine from age 50 in type 1 diabetes - Hepatitis B - Tetanus, Diphtheria, Pertussis (Tdap) if not up to date
Neoplasms: lymphoma, leukemia, myeloma, solid tumors treated with chemotherapy [87]	- Annual Influenza and Covid-19 - Pneumococcal PCV20 - Recombinant zoster vaccine from age 18 - Tetanus, Diphtheria, Pertussis (Tdap) if not up to date
Transplantation [88, 89]	**Solid Organ Transplantation** - Annual Influenza and Covid-19 - Pneumococcal PCV20 pretransplant and a booster after transplant - Poliovirus: 3 doses of inactivated vaccines 6–12 months after transplantation - Varicella: pre-transplantation if no evidence of varicella immunity - Recombinant zoster vaccine from age 18 - Hepatitis B pre-transplantation and at 12 months post-transplantation - Hepatitis A pre-transplantation and at 12 months post-transplantation - Measles, rumps and rubella (MMR) if not immune and pre-transplantation - Tetanus, Diphtheria, Pertussis (Tdap) if not up to date **Hematopoietic Stem Cell Transplant Recipients** - Annual Influenza and Covid-19 - Pneumococcal: 3 doses of PCV20, starting 3 months post-transplantation - *Haemophilus influenza* serotype b: 3 doses 6–12 months after transplantation - Hepatitis B: 3 doses 6–12 months after transplantation - Poliovirus: 3 doses of inactivated vaccines 6–12 months after transplantation - Varicella: 2 doses 24 months after transplantation if varicella seronegative - Recombinant zoster vaccine from age 18 - MMR if not immune and pre-transplantation - Tetanus, Diphtheria, Pertussis (Tdap) if not up to date
Immunosuppressive medication (including long-term systemic corticosteroids and radiotherapy) [90]	- Annual Influenza and Covid-19 - Pneumococcal PCV20 during maintenance therapy - Shingles (exclusively non-live vaccine) according to age - Tetanus, Diphtheria, Pertussis (Tdap) if not up to date
Human immunodeficiency virus [91]	- Annual Influenza and Covid-19 - Pneumococcal: Pneumococcal PCV20 - Shingles (exclusively non-live vaccine) ▪ from age 50 if CD4 ≥ 200 cells/ml ▪ from age 18 after immune reconstitution if CD4 < 200 cells/ml - Hepatitis A upon diagnosis - Hepatitis B upon diagnosis - Tetanus, Diphtheria, Pertussis (Tdap) if not up to date
Congenital or acquired immunodeficiency, common variable immunodeficiency (CVID), selective anti-polysaccharides antibody deficiency [92]	- Annual Influenza and Covid-19 - Pneumococcal PCV20 - Meningococcal (serogroups A, C, W, Y + serogroup B) - If T-cell deficit: Shingles (exclusively non-live vaccine) from age 18 - Tetanus, Diphtheria, Pertussis (Tdap) if not up to date
Complement deficiency (terminal or alternative pathway), mannose-binding lectin deficiency [93, 94]	- Annual Influenza and Covid-19 - Pneumococcal PCV20 - Meningococcal (serogroups A, C, W, Y + serogroup B) - Tetanus, Diphtheria, Pertussis (Tdap) if not up to date

Vaccination against respiratory syncytial virus (RSV) is recommended by the Centers for Diseases Control and Prevention for all adults aged 75 and older and for adults aged 60 to 74 at increased risk for severe RSV. Vaccination before the age of 60 must be evaluated on a case by case basis [95]

Adapted from [81, 84, 96]

### Immunoglobulins Replacement Therapy

Immunoglobulins replacement therapy (IGRT) plays a crucial role in reducing the risk of infections in patients with hypogammaglobulinemia, particularly those with PID. While a total serum IgG level below 4 g/L is often considered a strong indicator for further evaluation, the ESID diagnostic criteria emphasize a comprehensive assessment, including Ig levels, vaccine response, and clinical history. The decision to initiate IGRT should therefore be based not solely on IgG levels, but on the overall immunological and clinical context.

Studies in patients with CVID have shown that the risk of infections, particularly pneumonia, rises significantly when serum IgG levels fall below 3.0 g/L [97]. Furthermore, patients with persistent very low IgG levels (< 1.0 g/L) are generally considered at high risk for severe and potentially life-threatening infections. In such cases, IGRT is typically initiated even in the absence of a significant history of infections [98].

The consensus is that IGRT should aim to maintain trough serum IgG levels of at least 5.0 g/L [99]. Higher target levels (8–10 g/L) are advised for certain high-risk patient groups, such as those with bronchiectasis or chronic lung diseases [100]. In contrast, the role of IGRT in patients with hematologic malignancies and SOT recipients is less well established and remains controversial [53].

For selected patients with severe forms of IEI, curative approaches such as hematopoietic stem cell transplantation or gene therapy may be considered in specialized centers. While these modalities are beyond the scope of this review, they represent important therapeutic advances that complement supportive measures such as IGRT.

## Conclusion

Immunodeficiency in adults is more prevalent than commonly recognized and often remains underdiagnosed due to its heterogeneous presentation. This review highlights the most frequent causes of adult-onset immunodeficiency and provides a structured approach to its evaluation. Early recognition and appropriate workup are crucial, as delayed diagnosis may lead to recurrent, severe, or opportunistic infections with significant morbidity. It is essential to emphasize that a normal initial workup does not exclude underlying immunodeficiency, particularly in high-risk patients. Therefore, individuals with recurrent infections, poor vaccine responses, or other clinical features suggestive of immune dysfunction should be referred to an immunology specialist for comprehensive assessment and tailored management. A multidisciplinary approach involving primary care providers, immunologists, infectious diseases physicians, and other specialists is key to optimizing patient outcomes and preventing long-term complication.

## Data Availability

No datasets were generated or analysed during the current study.

## References

[1] Martinson ML, Lapham J (2024) Prevalence of immunosuppression among US adults. JAMA 331:880–882. 10.1001/jama.2023.28019 38358771 10.1001/jama.2023.28019 PMC10870224

[2] Seidel MG, Hauck F (2024) Multilayer concept of autoimmune mechanisms and manifestations in inborn errors of immunity: relevance for precision therapy. J Allergy Clin Immunol 153:615-628.e4. 10.1016/j.jaci.2023.12.022 38185417 10.1016/j.jaci.2023.12.022

[3] Seidel MG (2024) Rethinking PIDs: why the distinction between primary and secondary immune disorders is more frequently relevant than that between inborn and acquired errors of immunity. J Allergy Clin Immunol 153:1543–1545. 10.1016/j.jaci.2024.01.018 38316271 10.1016/j.jaci.2024.01.018

[4] Turvey SE, Biggs CM, James EL, Hildebrand KJ (2024) Should “primary immune disorder” replace “inborn error of immunity”? Names matter, but there is room for both. J Allergy Clin Immunol 153:1546–1547. 10.1016/j.jaci.2024.04.007 38649118 10.1016/j.jaci.2024.04.007

[5] Thalhammer J, Kindle G, Nieters A et al (2021) Initial presenting manifestations in 16,486 patients with inborn errors of immunity include infections and noninfectious manifestations. J Allergy Clin Immunol 148:1332-1341.e5. 10.1016/j.jaci.2021.04.015 33895260 10.1016/j.jaci.2021.04.015

[6] Moratti M, Conti F, Giannella M et al (2022) How to: diagnose inborn errors of intrinsic and innate immunity to viral, bacterial, mycobacterial, and fungal infections. Clin Microbiol Infect 28:1441–1448. 10.1016/j.cmi.2022.07.021 35934195 10.1016/j.cmi.2022.07.021

[7] ESID - European Society for Immunodeficiencies. https://esid.org/Working-Parties/Clinical-Working-Party/Resources/10-Warning-Signs-of-PID-General. Accessed 15 Apr 2024

[8] Jeffrey Model Foundation. In: info4pi.org. https://info4pi.org/. Accessed 15 Apr 2024

[9] Eldeniz FC, Gul Y, Yorulmaz A et al (2022) Evaluation of the 10 warning signs in primary and secondary immunodeficient patients. Front Immunol 13:900055. 10.3389/fimmu.2022.900055 35634313 10.3389/fimmu.2022.900055 PMC9136241

[10] Grammatikos AP, Tsokos GC (2012) Immunodeficiency and autoimmunity: lessons from systemic lupus erythematosus. Trends Mol Med 18:101–108. 10.1016/j.molmed.2011.10.005 22177735 10.1016/j.molmed.2011.10.005 PMC3278563

[11] Poli MC, Aksentijevich I, Bousfiha AA (2025) Human inborn errors of immunity: 2024 update on the classification from the International Union of Immunological Societies Expert Committee. J Hum Immunol 1:e20250003. 10.70962/jhi.20250003 10.70962/jhi.20250003 PMC12829761 41608114

[12] Rivière JG, Carot-Sans G, Piera-Jiménez J et al (2024) Development of an expert-based scoring system for early identification of patients with inborn errors of immunity in primary care settings – the PIDCAP project. J Clin Immunol 45:26. 10.1007/s10875-024-01825-3 39432052 10.1007/s10875-024-01825-3 PMC11493793

[13] Rider NL, Coffey M, Kurian A (2023) A validated artificial intelligence-based pipeline for population-wide primary immunodeficiency screening. J Allergy Clin Immunol 151:272–279. 10.1016/j.jaci.2022.10.005 36243223 10.1016/j.jaci.2022.10.005

[14] Staels F, Collignon T, Betrains A et al (2021) Monogenic adult-onset inborn errors of immunity. Front Immunol. 10.3389/fimmu.2021.753978 34867986 10.3389/fimmu.2021.753978 PMC8635491

[15] Bonilla FA, Barlan I, Chapel H et al (2016) International consensus document (ICON): common variable immunodeficiency disorders. The Journal of Allergy and Clinical Immunology: In Practice 4:38–59. 10.1016/j.jaip.2015.07.025 26563668 10.1016/j.jaip.2015.07.025 PMC4869529

[16] Resnick ES, Moshier EL, Godbold JH, Cunningham-Rundles C (2012) Morbidity and mortality in common variable immune deficiency over 4 decades. Blood 119:1650–1657. 10.1182/blood-2011-09-377945 22180439 10.1182/blood-2011-09-377945 PMC3286343

[17] Cunningham-Rundles C, Casanova J-L, Boisson B (2023) Genetics and clinical phenotypes in common variable immunodeficiency. Front Genet 14:1272912. 10.3389/fgene.2023.1272912 38274105 10.3389/fgene.2023.1272912 PMC10808799

[18] Christiansen M, Offersen R, Jensen JMB et al (2019) Identification of novel genetic variants in CVID patients with autoimmunity, autoinflammation, or malignancy. Front Immunol 10:3022. 10.3389/fimmu.2019.03022 32047491 10.3389/fimmu.2019.03022 PMC6996488

[19] Danieli MG, Murdaca G, Mezzanotte C et al (2025) Polyautoimmunity reflecting immune dysregulation in common variable immunodeficiency. Biomedicines 13:552. 10.3390/biomedicines13030552 40149529 10.3390/biomedicines13030552 PMC11940294

[20] Cinetto F, Scarpa R, Carrabba M et al (2021) Granulomatous lymphocytic interstitial lung disease (GLILD) in common variable immunodeficiency (CVID): a multicenter retrospective study of patients from Italian PID referral centers. Front Immunol 12:627423. 10.3389/fimmu.2021.627423 33777011 10.3389/fimmu.2021.627423 PMC7987811

[21] Barmettler S, Ong M-S, Farmer JR et al (2018) Association of immunoglobulin levels, infectious risk, and mortality with rituximab and hypogammaglobulinemia. JAMA Netw Open 1:e184169. 10.1001/jamanetworkopen.2018.4169 30646343 10.1001/jamanetworkopen.2018.4169 PMC6324375

[22] Settipane GA, Pudupakkam RK, McGowan JH (1978) Corticosteroid effect on immunoglobulins. J Allergy Clin Immunol 62:162–166. 10.1016/0091-6749(78)90101-x 681628 10.1016/0091-6749(78)90101-x

[23] Subesinghe S, Bechman K, Rutherford AI et al (2018) A systematic review and metaanalysis of antirheumatic drugs and vaccine immunogenicity in rheumatoid arthritis. J Rheumatol 45:733–744. 10.3899/jrheum.170710 29545454 10.3899/jrheum.170710

[24] Keven K, Sahin M, Kutlay S et al (2003) Immunoglobulin deficiency in kidney allograft recipients: comparative effects of mycophenolate mofetil and azathioprine. Transplant Infectious Dis 5:181–186. 10.1111/j.1399-3062.2003.00035.x 10.1111/j.1399-3062.2003.00035.x 14987202

[25] Baddley JW, Cantini F, Goletti D et al (2018) ESCMID Study Group for infections in compromised hosts (ESGICH) consensus document on the safety of targeted and biological therapies: an infectious diseases perspective (Soluble immune effector molecules [I]: anti-tumor necrosis factor-α agents). Clin Microbiol Infect Off Publ Eur Soc Clin Microbiol Infect Dis 24(Suppl 2):S10–S20. 10.1016/j.cmi.2017.12.025 10.1016/j.cmi.2017.12.025 29459143

[26] Cannon L, Pan A, Kovalick L et al (2023) Secondary immunodeficiencies and infectious considerations of biologic immunomodulatory therapies. Ann Allergy Asthma Immunol Off Publ Am Coll Allergy Asthma Immunol 130:718–726. 10.1016/j.anai.2023.02.010 10.1016/j.anai.2023.02.010 PMC10247415 36801438

[27] Ponsford M, Castle D, Tahir T et al (2019) Clozapine is associated with secondary antibody deficiency. Br J Psychiatry 214:83–89. 10.1192/bjp.2018.152 30259827 10.1192/bjp.2018.152 PMC6429246

[28] Wong JCY, Li PH (2020) Carbamazepine-induced B-cell aplasia: overlooked and overtreated. Ann Allergy Asthma Immunol Off Publ Am Coll Allergy Asthma Immunol 124:89–91. 10.1016/j.anai.2019.10.019 10.1016/j.anai.2019.10.019 31698095

[29] Travin M, Macris NT, Block JM, Schwimmer D (1989) Reversible common variable immunodeficiency syndrome induced by phenytoin. Arch Intern Med 149:1421–1422 2730260

[30] Svalheim S, Mushtaq U, Mochol M et al (2013) Reduced immunoglobulin levels in epilepsy patients treated with levetiracetam, lamotrigine, or carbamazepine. Acta Neurol Scand 127:11–15. 10.1111/ane.12044 10.1111/ane.12044 23190286

[31] George MD, Baker JF, Winthrop K et al (2020) Risk for serious infection with low-dose glucocorticoids in patients with rheumatoid arthritis: a cohort study. Ann Intern Med 173:870–878. 10.7326/M20-1594 32956604 10.7326/M20-1594 PMC8073808

[32] Wirsum C, Glaser C, Gutenberger S et al (2016) Secondary antibody deficiency in glucocorticoid therapy clearly differs from primary antibody deficiency. J Clin Immunol 36:406–412. 10.1007/s10875-016-0264-7 26980224 10.1007/s10875-016-0264-7

[33] Mikulska M, Lanini S, Gudiol C et al (2018) ESCMID study group for infections in compromised hosts (ESGICH) consensus document on the safety of targeted and biological therapies: an infectious diseases perspective (Agents targeting lymphoid cells surface antigens [I]: CD19, CD20 and CD52). Clin Microbiol Infect Off Publ Eur Soc Clin Microbiol Infect Dis 24(Suppl 2):S71–S82. 10.1016/j.cmi.2018.02.003 10.1016/j.cmi.2018.02.003 29447988

[34] Athni TS, Barmettler S (2023) Hypogammaglobulinemia, late-onset neutropenia, and infections following rituximab. Ann Allergy Asthma Immunol 130:699–712. 10.1016/j.anai.2023.01.018 36706910 10.1016/j.anai.2023.01.018 PMC10247428

[35] Elalouf A (2023) Infections after organ transplantation and immune response. Transpl Immunol 77:101798. 10.1016/j.trim.2023.101798 36731780 10.1016/j.trim.2023.101798

[36] Fernández-Ruiz M, Meije Y, Manuel O et al (2018) ESCMID Study Group for infections in compromised hosts (ESGICH) consensus document on the safety of targeted and biological therapies: an infectious diseases perspective (Introduction). Clin Microbiol Infect Off Publ Eur Soc Clin Microbiol Infect Dis 24(Suppl 2):S2–S9. 10.1016/j.cmi.2018.01.029 10.1016/j.cmi.2018.01.029 29427801

[37] Berbudi A, Rahmadika N, Tjahjadi AI, Ruslami R (2020) Type 2 diabetes and its impact on the immune system. Curr Diabetes Rev 16:442–449. 10.2174/1573399815666191024085838 31657690 10.2174/1573399815666191024085838 PMC7475801

[38] Shaikh SR, Beck MA, Alwarawrah Y, MacIver NJ (2024) Emerging mechanisms of obesity-associated immune dysfunction. Nat Rev Endocrinol 20:136–148. 10.1038/s41574-023-00932-2 38129700 10.1038/s41574-023-00932-2 PMC13082746

[39] Hasenmajer V, Sbardella E, Sciarra F et al (2020) The immune system in Cushing’s syndrome. Trends Endocrinol Metab 31:655–669. 10.1016/j.tem.2020.04.004 32387195 10.1016/j.tem.2020.04.004

[40] Bekker L-G, Beyrer C, Mgodi N et al (2023) HIV infection. Nat Rev Dis Primers 9:1–21. 10.1038/s41572-023-00452-3 37591865 10.1038/s41572-023-00452-3

[41] Mody A, Sohn AH, Iwuji C et al (2024) HIV epidemiology, prevention, treatment, and implementation strategies for public health. Lancet 403:471–492. 10.1016/S0140-6736(23)01381-8 38043552 10.1016/S0140-6736(23)01381-8

[42] Claudio P, Gabriella M (2023) Nephrotic syndrome: pathophysiology and consequences. J Nephrol 36:2179–2190. 10.1007/s40620-023-01697-7 37466816 10.1007/s40620-023-01697-7

[43] Otani IM, Lehman HK, Jongco AM et al (2022) Practical guidance for the diagnosis and management of secondary hypogammaglobulinemia: a work group report of the AAAAI primary immunodeficiency and altered immune response committees. J Allergy Clin Immunol 149:1525–1560. 10.1016/j.jaci.2022.01.025 35176351 10.1016/j.jaci.2022.01.025

[44] Ozen A, Lenardo MJ (2023) Protein-losing enteropathy. N Engl J Med 389:733–748. 10.1056/NEJMra2301594 37611123 10.1056/NEJMra2301594

[45] Vignes S, Bellanger J (2008) Primary intestinal lymphangiectasia (Waldmann’s disease). Orphanet J Rare Dis 3:5. 10.1186/1750-1172-3-5 18294365 10.1186/1750-1172-3-5 PMC2288596

[46] Cifarelli V, Eichmann A (2019) The intestinal lymphatic system: functions and metabolic implications. Cell Mol Gastroenterol Hepatol 7:503–513. 10.1016/j.jcmgh.2018.12.002 30557701 10.1016/j.jcmgh.2018.12.002 PMC6396433

[47] Na JE, Kim JE, Park S et al (2024) Experience of primary intestinal lymphangiectasia in adults: twelve case series from a tertiary referral hospital. World J Clin Cases 12:746–757. 10.12998/wjcc.v12.i4.746 38322684 10.12998/wjcc.v12.i4.746 PMC10841145

[48] Pearce J, Hadcocks L, Mansour S et al (2023) Profound and selective lymphopaenia in primary lymphatic anomaly patients demonstrates the significance of lymphatic-lymphocyte interactions. Front Immunol 14:1279077. 10.3389/fimmu.2023.1279077 38022535 10.3389/fimmu.2023.1279077 PMC10656747

[49] Liu T, Basseri S, Mussari B et al (2021) Generalized lymphatic anomalies and review of the current management landscape: a case report and review of the literature. J Med Case Rep 15:398. 10.1186/s13256-021-02953-9 34372919 10.1186/s13256-021-02953-9 PMC8353871

[50] Ivanovski I, Akbaroghli S, Pollazzon M et al (2018) Van Maldergem syndrome and Hennekam syndrome: further delineation of allelic phenotypes. Am J Med Genet A 176:1166–1174. 10.1002/ajmg.a.38652 29681106 10.1002/ajmg.a.38652

[51] Logan C, Koura D, Taplitz R (2020) Updates in infection risk and management in acute leukemia. Hematology 2020:135–139. 10.1182/hematology.2020000098 33275701 10.1182/hematology.2020000098 PMC7727589

[52] Tomblyn M, Chiller T, Einsele H et al (2009) Guidelines for preventing infectious complications among hematopoietic cell transplantation recipients: a global perspective. Biol Blood Marrow Transplant 15:1143–1238. 10.1016/j.bbmt.2009.06.019 19747629 10.1016/j.bbmt.2009.06.019 PMC3103296

[53] Kampouri E, Walti CS, Gauthier J, Hill JA (2022) Managing hypogammaglobulinemia in patients treated with CAR-T-cell therapy: key points for clinicians. Expert Rev Hematol 15:305–320. 10.1080/17474086.2022.2063833 35385358 10.1080/17474086.2022.2063833

[54] Allegra A, Tonacci A, Musolino C et al (2021) Secondary Immunodeficiency in Hematological Malignancies: Focus on Multiple Myeloma and Chronic Lymphocytic Leukemia. Front Immunol 12:738915. 10.3389/fimmu.2021.738915 34759921 10.3389/fimmu.2021.738915 PMC8573331

[55] Parikh SA, Leis JF, Chaffee KG et al (2015) Hypogammaglobulinemia in newly diagnosed chronic lymphocytic leukemia: natural history, clinical correlates, and outcomes. Cancer 121:2883–2891. 10.1002/cncr.29438 25931291 10.1002/cncr.29438 PMC4545721

[56] Kelleher P, Misbah SA (2003) What is Good’s syndrome? Immunological abnormalities in patients with thymoma. J Clin Pathol 56:12–16 12499426 10.1136/jcp.56.1.12 PMC1769851

[57] Tuano KS, Seth N, Chinen J (2021) Secondary immunodeficiencies: an overview. Ann Allergy Asthma Immunol 127:617–626. 10.1016/j.anai.2021.08.413 34481993 10.1016/j.anai.2021.08.413

[58] Chinen J, Shearer WT (2010) Secondary immunodeficiencies, including HIV infection. J Allergy Clin Immunol 125:S195-203. 10.1016/j.jaci.2009.08.040 20042227 10.1016/j.jaci.2009.08.040 PMC6151868

[59] Cohen G (2020) Immune dysfunction in uremia 2020. Toxins 12:439. 10.3390/toxins12070439 32635646 10.3390/toxins12070439 PMC7404977

[60] Espi M, Koppe L, Fouque D, Thaunat O (2020) Chronic kidney disease-associated immune dysfunctions: impact of protein-bound uremic retention solutes on immune cells. Toxins 12:300. 10.3390/toxins12050300 32384617 10.3390/toxins12050300 PMC7291164

[61] Albillos A, Martin-Mateos R, Van der Merwe S et al (2022) Cirrhosis-associated immune dysfunction. Nat Rev Gastroenterol Hepatol 19:112–134. 10.1038/s41575-021-00520-7 34703031 10.1038/s41575-021-00520-7

[62] Long B, Koyfman A, Gottlieb M (2021) Complications in the adult asplenic patient: a review for the emergency clinician. Am J Emerg Med 44:452–457. 10.1016/j.ajem.2020.03.049 32247651 10.1016/j.ajem.2020.03.049

[63] Giulietti G, Zama D, Conti F et al (2022) In-depth immunological typization of children with sickle cell disease: a preliminary insight into its plausible correlation with clinical course and hydroxyurea therapy. J Clin Med 11:3037. 10.3390/jcm11113037 35683425 10.3390/jcm11113037 PMC9181704

[64] Cavalcanti NV, Palmeira P, Jatene MB et al (2021) Early thymectomy is associated with long-term impairment of the immune system: a systematic review. Front Immunol 12:774780. 10.3389/fimmu.2021.774780 34899730 10.3389/fimmu.2021.774780 PMC8656688

[65] Terrones-Campos C, Ledergerber B, Vogelius IR et al (2019) Lymphocyte count kinetics, factors associated with the end-of-radiation-therapy lymphocyte count, and risk of infection in patients with solid malignant tumors treated with curative-intent radiation therapy. Int J Radiat Oncol Biol Phys 105:812–823. 10.1016/j.ijrobp.2019.07.013 31344435 10.1016/j.ijrobp.2019.07.013

[66] Filomena CA, Filomena AP, Hudock J, Ballas SK (1992) Evaluation of serum immunoglobulins by protein electrophoresis and rate nephelometry before and after therapeutic plasma exchange. Am J Clin Pathol 98:243–248. 10.1093/ajcp/98.2.243 1510038 10.1093/ajcp/98.2.243

[67] Howard J, Huang A, Li Z et al (2021) An evidence review of face masks against COVID-19. Proc Natl Acad Sci USA 118:e2014564118. 10.1073/pnas.2014564118 33431650 10.1073/pnas.2014564118 PMC7848583

[68] Maertens JA, Girmenia C, Brüggemann RJ, et al (2018) European guidelines for primary antifungal prophylaxis in adult haematology patients: summary of the updated recommendations from the European Conference on Infections in Leukaemia. J Antimicrob Chemother 73:3221–3230. 10.1093/jac/dky286 10.1093/jac/dky286 30085172

[69] National Institutes of Health, HIV Medicine Association, and Infectious Diseases Society of Guidelines for the Prevention and Treatment of Opportunistic Infections in Adults and Adolescents with HIV. https://clinicalinfo.hiv.gov/en/guidelines/adult-and-adolescent-opportunistic-infection. Accessed 21 Feb 2025

[70] Ullmann AJ, Schmidt-Hieber M, Bertz H et al (2016) Infectious diseases in allogeneic haematopoietic stem cell transplantation: prevention and prophylaxis strategy guidelines 2016. Ann Hematol 95:1435–1455. 10.1007/s00277-016-2711-1 27339055 10.1007/s00277-016-2711-1 PMC4972852

[71] Malpica L, van Duin D, Moll S (2019) Preventing infectious complications when treating non-malignant immune-mediated hematologic disorders. Am J Hematol 94:1396–1412. 10.1002/ajh.25642 31571266 10.1002/ajh.25642

[72] Winthrop KL, Mariette X, Silva JT et al (2018) ESCMID Study Group for Infections in compromised hosts (ESGICH) consensus document on the safety of targeted and biological therapies: an infectious diseases perspective (Soluble immune effector molecules [II]: agents targeting interleukins, immunoglobulins and complement factors). Clin Microbiol Infect 24:S21–S40. 10.1016/j.cmi.2018.02.002 29447987 10.1016/j.cmi.2018.02.002

[73] Pagano L, Maschmeyer G, Lamoth F, et al (2025) Primary antifungal prophylaxis in hematological malignancies. Updated clinical practice guidelines by the European Conference on Infections in Leukemia (ECIL). Leukemia. 10.1038/s41375-025-02586-7 10.1038/s41375-025-02586-7 PMC12208874 40200079

[74] Maertens J, Lodewyck T, Donnelly JP et al (2023) Empiric vs preemptive antifungal strategy in high-risk neutropenic patients on fluconazole prophylaxis: a randomized trial of the european organization for research and treatment of cancer. Clin Infect Dis Off Publ Infect Dis Soc Am 76:674–682. 10.1093/cid/ciac623 10.1093/cid/ciac623 PMC9938744 35906831

[75] Baden LR, Swaminathan S, Angarone M et al (2016) Prevention and treatment of cancer-related infections, Version 2.2016, NCCN Clinical Practice Guidelines in Oncology. J Natl Compr Cancer Netw JNCCN 14:882–913. 10.6004/jnccn.2016.0093 27407129 10.6004/jnccn.2016.0093

[76] Kotton CN, Kumar D, Manuel O et al (2025) The Fourth international consensus guidelines on the management of cytomegalovirus in solid organ transplantation. Transplantation. 10.1097/TP.0000000000005374 40200403 10.1097/TP.0000000000005374 PMC12180710

[77] Ljungman P, Alain S, Chemaly RF et al (2025) Recommendations from the 10th European conference on infections in leukaemia for the management of cytomegalovirus in patients after allogeneic haematopoietic cell transplantation and other T-cell-engaging therapies. Lancet Infect Dis 25:e451–e462. 10.1016/S1473-3099(25)00069-6 40188837 10.1016/S1473-3099(25)00069-6

[78] Lau GKK (2008) Hepatitis B reactivation after chemotherapy: two decades of clinical research. Hepatol Int 2:152–162. 10.1007/s12072-008-9056-3 19669300 10.1007/s12072-008-9056-3 PMC2716860

[79] Shih C-A, Chen W-C, Yu H-C et al (2015) Risk of severe acute exacerbation of chronic HBV infection cancer patients who underwent chemotherapy and did not receive anti-viral prophylaxis. PLoS ONE 10:e0132426. 10.1371/journal.pone.0132426 26274393 10.1371/journal.pone.0132426 PMC4537229

[80] US Preventive Services Task Force (2023) Screening for latent tuberculosis infection in adults: US preventive services task force recommendation statement. JAMA 329:1487–1494. 10.1001/jama.2023.4899 37129649 10.1001/jama.2023.4899

[81] Rubin LG, Levin MJ, Ljungman P et al (2014) Executive summary: 2013 IDSA clinical practice guideline for vaccination of the immunocompromised host. Clin Infect Dis 58:309–318. 10.1093/cid/cit816 24421306 10.1093/cid/cit816

[82] Alukal JJ, Naqvi HA, Thuluvath PJ (2022) Vaccination in chronic liver disease: an update. J Clin Exp Hepatol 12:937–947. 10.1016/j.jceh.2021.12.003 34975241 10.1016/j.jceh.2021.12.003 PMC8710401

[83] Bonanni P, Grazzini M, Niccolai G et al (2016) Recommended vaccinations for asplenic and hyposplenic adult patients. Hum Vaccines Immunother 13:359–368. 10.1080/21645515.2017.1264797 10.1080/21645515.2017.1264797 PMC5328222 27929751

[84] Ma BM, Yap DYH, Yip TPS et al (2021) Vaccination in patients with chronic kidney disease—review of current recommendations and recent advances. Nephrology 26:5–11. 10.1111/nep.13741 32524684 10.1111/nep.13741

[85] Khwaja A (2012) KDIGO clinical practice guidelines for acute kidney injury. Nephron Clin Pract 120:c179-184. 10.1159/000339789 22890468 10.1159/000339789

[86] Mastrovito B, Lardon A, Dubromel A et al (2024) Understanding the gap between guidelines and influenza vaccination coverage in people with diabetes: a scoping review. Front Public Health 12:1360556. 10.3389/fpubh.2024.1360556 38706547 10.3389/fpubh.2024.1360556 PMC11066301

[87] Tsigrelis C, Ljungman P (2016) Vaccinations in patients with hematological malignancies. Blood Rev 30:139–147. 10.1016/j.blre.2015.10.001 26602587 10.1016/j.blre.2015.10.001

[88] Viganò M, Beretta M, Lepore M et al (2023) Vaccination recommendations in solid organ transplant adult candidates and recipients. Vaccines 11:1611. 10.3390/vaccines11101611 37897013 10.3390/vaccines11101611 PMC10611006

[89] Reynolds G, Hall VG, Teh BW (2023) Vaccine schedule recommendations and updates for patients with hematologic malignancy post-hematopoietic cell transplant or CAR T-cell therapy. Transpl Infect Dis 25(S1):e14109. 10.1111/tid.14109 37515788 10.1111/tid.14109 PMC10909447

[90] Alnaimat F, Sweis JJG, Jansz J et al (2023) Vaccination in the era of immunosuppression. Vaccines 11:1446. 10.3390/vaccines11091446 37766123 10.3390/vaccines11091446 PMC10537746

[91] De Vito A, Colpani A, Trunfio M et al (2023) Living with HIV and getting vaccinated: a narrative review. Vaccines 11:896. 10.3390/vaccines11050896 37243000 10.3390/vaccines11050896 PMC10220625

[92] Bonilla FA (2018) Update: vaccines in primary immunodeficiency. J Allergy Clin Immunol 141:474–481. 10.1016/j.jaci.2017.12.980 29288077 10.1016/j.jaci.2017.12.980

[93] Centers for Disease Control and Prevention (CDC) (2011) Updated recommendations for use of meningococcal conjugate vaccines –- advisory Committee on Immunization Practices (ACIP), 2010. MMWR Morb Mortal Wkly Rep 60:72–76 21270745

[94] Folaranmi T, Rubin L, Martin SW et al (2015) Use of serogroup B Meningococcal vaccines in persons aged ≥10 years at increased risk for serogroup B meningococcal disease: recommendations of the advisory committee on immunization practices, 2015. MMWR Morb Mortal Wkly Rep 64:608–612 26068564 PMC4584923

[95] CDC (2025) RSV Vaccines. In: Respir. Syncytial Virus Infect. RSV. https://www.cdc.gov/rsv/vaccines/index.html. Accessed 10 Jul 2025

[96] Gerber C, Bart P-A, Comte D (2023) [Should we suspect immune deficiency in internal medicine hospitalized patients?]. Rev Med Suisse 19:2200–2205. 10.53738/REVMED.2023.19.851.2200 10.53738/REVMED.2023.19.851.2200 37994599

[97] Bayrakci B, Ersoy F, Sanal O et al (2005) The efficacy of immunoglobulin replacement therapy in the long-term follow-up of the B-cell deficiencies (XLA, HIM, CVID). Turk J Pediatr 47:239–246 16250308

[98] Agarwal S, Cunningham-Rundles C (2007) Assessment and clinical interpretation of reduced IgG values. Ann Allergy Asthma Immunol Off Publ Am Coll Allergy Asthma Immunol 99:281–283. 10.1016/S1081-1206(10)60665-5 10.1016/S1081-1206(10)60665-5 PMC3099256 17910333

[99] Orange JS, Hossny EM, Weiler CR et al (2006) Use of intravenous immunoglobulin in human disease: a review of evidence by members of the primary immunodeficiency committee of the American Academy of Allergy, Asthma and Immunology. J Allergy Clin Immunol 117:S525–S553. 10.1016/j.jaci.2006.01.015 16580469 10.1016/j.jaci.2006.01.015

[100] Wall LA, Wisner EL, Gipson KS, Sorensen RU (2020) Bronchiectasis in Primary antibody deficiencies: a multidisciplinary approach. Front Immunol 11:522. 10.3389/fimmu.2020.00522 32296433 10.3389/fimmu.2020.00522 PMC7138103

